# Primary Repair versus Reconstruction in Patients with Bilateral Anterior Cruciate Ligament Injuries: What Do Patients Prefer?

**DOI:** 10.1155/2022/3558311

**Published:** 2022-09-13

**Authors:** Harmen D. Vermeijden, Edoardo Monaco, Fabio Marzilli, Xiuyi A. Yang, Jelle P. van der List, Andrea Ferretti, Gregory S. DiFelice

**Affiliations:** ^1^Hospital for Special Surgery, Department of Orthopaedic Surgery, New York, NY, USA; ^2^Spaarne Gasthuis Hospital, Department of Orthopaedic Surgery, Hoofddorp, Netherlands; ^3^Amsterdam UMC, University of Amsterdam, Amsterdam Movement Science, Department of Orthopaedic Surgery, Amsterdam, Netherlands; ^4^Orthopaedic Unit and Kirk Kilgour Sports Injury Centre, S. Andrea Hospital, University of Rome La Sapienza, Rome, Italy

## Abstract

**Purpose:**

The purpose is to evaluate knee preference and functional outcomes of patients with primary anterior cruciate ligament (ACL) repair in one knee and ACL reconstruction in the contralateral side.

**Methods:**

All patients who underwent both procedures were retrospectively reviewed at minimum two-year follow-up. Patients were asked to complete questionnaires regarding their operated knees' preferences during rehabilitation, daily activities, sports activities, and overall function. Furthermore, the Subjective International Knee Documentation Committee, Forgotten Joint Score-12, and Anterior Cruciate Ligament-Return to Sport after Injury were completed.

**Results:**

Twenty-one patients were included. All patients underwent ACL reconstruction first, which was displayed at younger age at surgery (24 vs. 33 years, *p* = 0.010) and longer follow-up (10.2 vs. 2.3 years, *p* < 0.001), respectively. Thirty-three percent preferred the repaired knee, 11% the reconstructed knee, and 56% had no preference; however, 78% indicated that their repaired knee was less painful during rehabilitation and 83% reported earlier range of motion (ROM) return following repair, which was similar for both knees in 17%. Eighty-three percent of patients indicated better function and progression during rehabilitation with their repaired knee and 11% with their reconstructed knees. No statistical differences were found in patient-reported outcomes between both procedures (all *p* > 0.4). Objective laxity assessment showed mean side-to-side difference of 0.6 mm between both sides in favor of the reconstructed knee.

**Conclusion:**

This study showed that ACL repair and ACL reconstruction lead to similar functional outcomes. However, patients undergoing both procedures may have less pain, earlier ROM return, and faster rehabilitation progression following primary repair.

## 1. Introduction

Anterior cruciate ligament (ACL) tears represent one of the most common sports-medicine injuries, with an incidence of 200,000 in the United States per year [1, 2]. As the most important restraint to anterior tibial translation (ATT), ligament reconstruction has been advocated in the ACL deficient knee, especially for those aiming to return to highly competitive and pivoting sports [3]. In recent years, however, advancements in appropriate patient selection criteria, minimally invasive surgical techniques, and modern rehabilitation protocols have led to a resurgence of interest in arthroscopic primary ACL repair [4, 5]. This procedure may be a less morbid surgical alternative for those patients presenting with functional impairment after acute proximal ACL injuries [6, 7], although it should be noted that high-quality evidence for ACL repair is still limited given its recent resurgence [8].

Compared with ACL reconstruction, primary ACL repair is associated with some potential benefits as the native ligament and its proprioceptive properties can be preserved while avoiding donor-site morbidity, thereby potentially allowing an easier postoperative rehabilitation [9, 10]. Furthermore, primary repair preserves the possibility of performing an ACL reconstruction if the repaired ACL fails [11]. On the contrary, it has been suggested that primary repair failure rates are higher than those reported in the ACL reconstruction literature [12]. Although recent studies have shown improved functional outcomes of primary ACL repair as compared to ACL reconstruction [13], there has always been a concern of selection bias because repair patients seem to be older and participate at lower activity levels, respectively [6, 14].

Therefore, the purpose of this study was to evaluate knee preference and functional outcomes of a group of patients with a primary anterior cruciate ligament (ACL) repair on one knee and ACL reconstruction on the contralateral side, which allows for direct comparative analysis of both procedures and correction for potential confounders when analyzing different patient cohorts. The hypothesis was that patients would prefer their repaired knee over their reconstructed knee at short-term follow-up based on the less invasive nature of the surgery. Furthermore, we hypothesized that patient-reported outcome measures (PROMs) would be superior in the repaired over the reconstructed knee.

## 2. Methods

### 2.1. Patient Selection

This IRB approved study (IRB number: 2017-0404-CR2) retrospectively reviewed all patients treated with arthroscopic primary ACL repair on one knee and ACL reconstruction on the other knee in the prospective databases of two sports-medicine surgeons (AF and GSD) at two centers in different countries (USA and Italia). All patients were treated between April 2008 and December 2019 using the same surgical intraoperative treatment algorithm: patients with proximal tears with good to excellent tissue quality underwent ACL repair while a standard ACL reconstruction was performed otherwise [15, 16] At the time of study initiation, 26 patients were identified that underwent both procedures on either knee of which other knee surgeons had performed ACL reconstruction in 22 of the 26 patients. It should be noted that both surgeries were not performed simultaneously. Patients were considered for inclusion if a minimum of two-year follow-up for each operated knee was present and were excluded when follow-up was insufficient (*n* = 1). As a result, 25 patients met the criteria and were contacted to complete an outcome questionnaire to assess which knee they preferred. Among these patients, four did not complete the questionnaire or could not be contacted. Therefore, 21 patients were ultimately enrolled in this study.

### 2.2. Surgical Techniques

The surgical technique selected was at the discretion of the treating surgeon, which has been previously described in the literature in more detail [17, 18]. First, anesthesia was administered based upon anesthesiologist and patient preferences. Then, the patient was placed in the supine position, and the operative leg was prepped and draped as for standard knee arthroscopy. Both surgeons subsequently performed selective arthroscopic primary ACL repair in patients with proximal avulsion tears only, in which the native ligament was reapproximated towards the femoral insertion site. AF performed arthroscopic single transosseous femoral tunnel repair in which the torn ligament was sutured through the use of a lasso-loop knot-tying configuration, while GSD performed arthroscopic dual suture-anchor fixation repair whereby the sutures were passed in a Bunnell-pattern through each bundle of the distal remnant. Furthermore, it should be noted that availability of a new augmentation method (InternalBrace; Arthrex, Naples, FL) clinically began in 2015, and then became the standard of care in all patients treated by GSD, as previously described more extensively [19].

**Figure 1:**
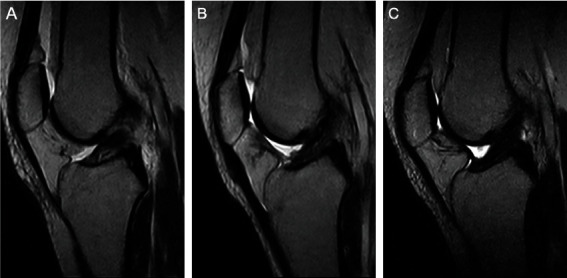
Pre- and postoperative MRI images of a patient treated with primary ACL repair using transosseous tunnel technique. (a) A sagittal T1-weighted MRI image of a proximal type I tear with excellent tissue quality is seen. (b) A sagittal T1-weighted MRI image at six months after surgery. (c) A sagittal T1-weighted MRI image at one year after surgery.

It should be noted that the major difference between both techniques was the utilized fixation method, which was either performed via a transosseous tunnel technique and tying the sutures over a bone bridge with a button by AF (eight patients) or using knotless suture anchors by GSD (13 patients Figure 1).

ACL reconstructions were performed using a variety of different grafts and techniques. Most patients underwent autograft reconstruction (six patients' bone-patellar tendon-bone (BPTB) autograft, twelve hamstring autografts, and one quadriceps autograft), whereas two underwent soft tissue allografts.

### 2.3. Rehabilitation Protocols

Similar to the surgical techniques, rehabilitation protocols were also at the discretion of the treating surgeon, but generally the postoperative rehabilitation was similar. All patients received a knee brace for the first four to six postoperative weeks. Immediate weight-bearing was allowed as tolerated by the patient. Passive range of motion (ROM) exercises, swelling control, and weight-bearing were initiated within the initial days after surgery. At four to six weeks, patients started a supervised strengthening program under a physical therapist's guidance. Return to sports (RTS) activities was permitted at minimum six months postoperatively, and based upon sport-specific assessments.

### 2.4. Outcome Measures

Clinical assessment was performed via Lachman test and pivot shift test, while objective laxity measurements were assessed using either KT-1000 arthrometer (MEDmetric Corp, San Diego, CA, USA) or Rolimeter arthrometer (Aircast, Germany). In addition, all adverse events were documented and recorded. Therefore, clinical failures were assessed, defined as grade ≥2 Lachman, and/or grade ≥2 pivot shift test, side-to-side difference ≥3 mm, or symptomatic subjective feeling of instability. Furthermore, patients were asked if any surgical intervention, besides revision surgery, had been performed.

For comparative analysis, a 10-question questionnaire was developed to assess which operated knee patients preferred during rehabilitation, daily activities, sports activities, and overall function (Appendix), which were completed either in clinic or via mail. Finally, functional outcomes after surgery were assessed using the Forgotten-Joint-Score-12 (FJS-12) [20], International Knee Documentation Committee (IKDC) Subjective form, 20 and Anterior Cruciate Ligament-Return to Sport after Injury (ACL-RSI) Scale (short-version) [20].

For final analysis, only patients with intact ACL repairs and grafts were included. Subgroups were defined based upon age at latest surgery (<30 and ≥ 30 years) [21], gender, BMI at latest surgery (<25 and ≥ 25 kg/m^2^) [22], and surgical delay of the repair procedure (<3 and ≥ 3 weeks post-injury) [23].

### 2.5. Statistical Analysis

All statistical analyses were performed with SPSS, version 26 (IBM Corp., Armonk, NY). Continuous data were presented in mean with their ranges and compared using independent *t*-test, while categorical variables were presented in absolute frequency (*n*) and relative frequency (%) and compared using Chi-square tests (or Fisher's exact test in case one of the numbers is < 5). A descriptive analysis was conducted to assess knee preference. Significance of statistical differences was attributed to *p*-value of <0.05.

## 3. Results

### 3.1. Patient Demographics

Twenty-one patients were included in this study. Mean age was 28 years (range 14–51 years), 38% were male, mean BMI was 23.8 kg/m^2^ (range 18.8–31.3 kg/m^2^), mean follow-up was 6.3 years (range 2.0–20.8 year), and 50% of patients underwent right-sided knee repair surgery. Thirteen patients (63%) underwent suture-anchor fixation repair, which included nine Internal Brace augmented repairs (43%), while eight (38%) were treated using a transosseous femoral tunnel technique without augmentation. Since all patients had their ACL reconstruction procedure first (explained by the recent introduction of arthroscopic ACL repair), patients were younger at time of their surgery (24 years vs. 33 years, *p*=0.010) and had longer follow-up (10.2 years vs. 2.3 years, *p* < 0.001) for their reconstructed knee, respectively. Baseline demographics are further detailed in Table 1.

### 3.2. Adverse Events

At final follow-up, there was one failure among the repaired knees at 1.4 years after surgery (5%) and two failures (10%) among the reconstructed knees (one BPTB at 1.1 years after surgery and one hamstring autograft at 2.3 years after surgery). The failed repair was subsequently converted into an uncomplicated ACL reconstruction using quadriceps autograft, and the reconstructed knees both underwent uncomplicated one-stage ACL revision reconstruction using one BPTB and one hamstring autograft, respectively. One of the failed reconstructions had an additional meniscus lesion that required a partial meniscectomy. No other complications were recorded.

### 3.3. Knee Preferences

Of all patients without failure, 78% indicated that their repaired knee was less painful during rehabilitation versus 22% of the reconstructed knees. In addition, 83% of patients reported that their repaired knee had earlier ROM return, as opposed to 0% of the reconstructed knees. Eighty-three percent of patients indicated that the rehabilitation following repair was advanced more rapidly than their reconstructed knee, as opposed to 11% who reported better function and progression during rehabilitation following their ACL reconstruction, and 6% did not note a difference. Finally, when asked which knee was their better knee, 33% of patients preferred the repaired knee, 11% the reconstructed knee, and 56% had no preferences (Table 2).

### 3.4. Subgroup Analyses

When comparing subgroups, no significant differences were noted between both genders in any of the survey domains (all *p* > 0.05). Similarly, no significant differences were found in any of the 10 domains between patients younger and older than 30 years, treated within and after 3 weeks post-injury, nor in those with BMI above and below 25 kg/m^2^ (all *p* > 0.05).

## 4. Clinical Assessment

All patients that returned for clinical evaluation and without failure (*n* = 18; 86%; three patients could not be seen back clinically due to COVID-19) achieved full ROM of their repaired knee. Among these patients, the Lachman test was negative in 15 and grade 1 with a firm endpoint in three. Furthermore, the pivot shift test was negative in 17 patients and grade 1 in one patient. Objective laxity assessments were available in 12 patients and showed a mean manual side-to-side difference of 0.6 mm (range −2.7–3.0 mm) in favor of the reconstructed knee, without any patients having more than 3 mm difference between both sides.

### 4.1. Patient-Reported Outcome Measurements

When comparing functional outcomes between the intact repaired and intact reconstructed knees, mean IKDC subjective score was 89.3 ± 8.3 vs. 87.5 ± 13.8, FJS-12 score was 87.0 ± 11.8 vs. 81.9 ± 23.8, and ACL-RSI score was 73.6 ± 21.8 vs. 73.5 ± 28.0. No significant differences were found in any of these outcomes following both procedures (all *p* > 0.4; Table 3).

## 5. Discussion

The most important finding of this study was that most patients preferred the repaired knee over their reconstructed knee during all aspects of the rehabilitation phase. In addition, it was noted that similar functional outcomes could be achieved following both surgical procedures at short-term follow-up. Therefore, primary ACL repair can result in similar short-term outcomes compared to ACL reconstruction, but the repair procedure seems to be more frequently associated with an easier rehabilitation experience.

This is the first study assessing functional outcomes following primary ACL repair and ACL reconstruction in the same patient. When asking patients which knee they preferred overall, it was noted that ten patients had no preferences (56%), six preferred their repaired knee (33%), while only two preferred their reconstructed knee (11%). However, most patients preferred their repaired knee over their reconstructed knee during all aspects of rehabilitation, including pain, return of ROM, swelling, and recovery progression. The less invasive nature of repair surgery likely plays an important role in these differences. Several authors have advocated that this procedure is considerably less invasive as the native ligament can be preserved, donor-site morbidity can be avoided, and only small tunnels need to be drilled [6]. Furthermore, preservation of the ACL may have additional benefits as proprioceptive function may be maintained. As a result, this likely contributes to this treatment's potential inherent advantages over ACL reconstruction, as seen in this study.

Given the differences in surgical morbidity, it was noted that 83% of patients reported that ROM patients returned earlier following their repair surgery than following the ACL reconstruction procedure, while the remaining 17% did not experience a difference. In a recent case-control study performed by our group, we have previously shown that repair patients have improved ROM and tends towards fewer complications than reconstruction (references blinded for review purposes), which often leads to a faster postoperative recovery progression. However, it is important to note that rehabilitation and return-to-play protocols have not yet been clearly established for this procedure [12]. This has also been highlighted in a recent survey, which showed that current rehabilitation protocols varied considerably among surgeons utilizing primary repair techniques [24]. Nevertheless, one may indeed expect that the rehabilitation following primary repair is significantly less painful, complicated, and faster than those following reconstruction given the absence of graft harvesting and drilling of large tunnels.

Data in this study showed equivalent patient-reported outcomes between the repaired and reconstructed knees. A recent meta-analysis similarly reported that functional outcome scores of >85% of maximum scores could be expected following primary ACL repair [6]. When looking at comparative studies, two recent level I studies also did not find statistical differences in subjective outcomes between patients treated with dynamic augmented ACL suture repair and ACL reconstruction [25, 26]. Given these findings, it appears that patients treated with repair seem to have similar functional outcomes than those treated with reconstruction.

When looking closely at the functional outcomes, patients had less joint awareness (i.e. higher FJS-12 scores) in their repaired knee as compared to their reconstructed one (5.1-point difference), but this difference did not reach significance. Our group has previously shown that repair patients have significantly less joint awareness (indicated by higher FJS-12 scores) than those treated with ACL reconstruction (10.7-point difference) (references blinded for review purposes). It has been suggested that the less invasive in nature of repair surgery, in which the native ligament can be preserved while avoiding donor-site morbidity, may contribute to decreased daily knee joint awareness following primary repair as compared with reconstruction [13]. When compared to other patient-reported outcomes, it has been shown that the FJS-12 has less ceiling effect [27], thereby allowing improved discrimination in well-performing patients. Therefore, it may be possible that potential functional outcome differences between repaired and reconstructed knees may not be detected with other outcome metrics. Future prospective studies need to confirm if there is indeed a difference in joint awareness between both procedures, especially as the current study was obviously underpowered for these analyses.

This study found no difference in patient-reported knee stability during sports activities in patients who underwent both surgical procedures. In addition, psychological readiness to return to sport after surgery was similar at short-term follow-up following both primary ACL repair and ACL reconstruction (73.6 ± 21.8 vs. 73.5 ± 28.0). When reviewing the literature, the optimal ACL-RSI score threshold of returning to preinjury sports level following ACL reconstruction at 2-years is ≥ 65 [28], although this threshold has not been established for primary repair. Given these findings, however, this study suggests that both procedures lead to psychological readiness to return to sports participation, which is one of the general goals for ACL surgery.

### 5.1. Limitations

Obviously, there are limitations to this study. First, this study is limited by the relatively small sample size, which can be explained by the relatively low incidence of bilateral ACL injuries and proximal tear location [29]. Secondly, the differences in age at time of surgery and length of follow-up between both procedures may have influenced the subjective outcomes in this study. Furthermore, this could also have introduced possible recall bias as patients may forget their experiences after their first surgery. In addition, the repaired ACLs were only followed up until the short term (range 2.0 to 3.7 years), and these patients should be followed up at least until the mid-term. Furthermore, it is important to note that data regarding delay between injury and surgery and concomitant lesions were missing in a large subset of patients (these patients were treated by different surgeons in other hospitals first), which could therefore not be reported. However, this may have significantly influenced the outcomes. Finally, the utilization of different surgical techniques and rehabilitation protocols might also influence the present results. On the contrary, however, this could have increased the generalizability of the study findings.

## 6. Conclusion

This study showed that ACL repair and ACL reconstruction lead to similar functional outcomes at short-term follow-up. Primary repair, however, was associated with less pain, earlier return of ROM, faster progression during rehabilitation, and overall subjective better function as experienced by patients undergoing both procedures. Patients should be counseled regarding the risks and benefits of both surgical procedures.

## Figures and Tables

**Table 1:** Patient demographics of patients treated with primary ACL repair and ACL reconstruction.

	Primary repair	Reconstruction	*P* value
Age (years); mean ± SD	33.1 ± 11.6	24.2 ± 10.0	**0.010**
Male gender; freq. (%)	8 (38%)	8 (38%)	N/A
First surgery; freq. (%)	0 (0%)	21 (100%)	**<0.001**
Right side; freq. (%)	9 (43%)	12 (57%)	0.355
Follow-up (years); ±SD	1.9 ± 0.8	10.3 ± 5.9	**<0.001**

N/A indicates not applicable.

**Table 2:** Preferences between both knees following primary ACL repair and ALC reconstruction in the same patient^*∗*^.

	Primary repair	Reconstruction	No preferences
Which knee had less pain postoperatively?	13 (72%)	5 (28%)	0 (0%)
Which knee was less painful during rehabilitation?	14 (78%)	4 (22%)	0 (0%)
Which knee had earlier return of ROM?	15 (83%)	0 (0%)	3 (17%)
Which knee was less swollen during rehabilitation?	13 (72%)	1 (6%)	4 (22%)
Which knee had better function and progressed faster during rehabilitation?	15 (83%)	2 (11%)	1 (6%)
Which knee feels better during daily activities?	6 (33%)	1 (6%)	11 (61%)
Which knee feels better during stair-climbing?	2 (11%)	4 (22%)	12 (67%)
Which knee feels more stable during sporting activities?	2 (11%)	3 (17%)	13 (72%)
Which knee are you currently more confident about during sporting activities?	3 (17%)	5 (28%)	10 (56%)
Which knee is your better knee overall?	6 (33%)	2 (11%)	10 (56%)

Asterisk indicated reported in number (%); ROM, range of motion.

**Table 3:** Patient-reported outcomes of those treated with primary ACL repair and ACL reconstruction.

	Primary repair	Reconstruction	*P* value
Subjective IKDC; mean ± SD	89.3 ± 8.3	87.5 ± 13.8	0.646
FJS-12; mean ± SD	87.0 ± 11.8	81.9 ± 23.8	0.426
ACL-RSI; mean ± SD	73.6 ± 21	73.5 ± 28.0	0.993

IKDC indicates International Knee Documentation Committee; FJS-12, forgotten joint Score-12; ACL-RSI, anterior cruciate ligament return to sport after injury.

## Data Availability

Data are available on request.

## Appendix

Please answer the following 10 questions regarding preferences between both knees.

1. Which knee had less pain postoperatively?○ repaired knee ○ reconstructed knee ○ no difference
2. Which knee was less painful during rehabilitation? ○ repaired knee ○ reconstructed knee ○ no difference
3. Which knee had an earlier return of range of motion? ○ repaired knee ○ reconstructed knee ○ no difference
4. Which knee was less swollen during rehabilitation? ○ repaired knee ○ reconstructed knee ○ no difference
5. Which knee had better function and progressed faster during rehabilitation? ○ repaired knee ○ reconstructed knee ○ no difference
6. Which knee feels better during daily activities? ○ repaired knee ○ reconstructed knee ○ no difference
7. Which knee feels better during stair-climbing? ○ repaired knee ○ reconstructed knee ○ no difference
8. Which knee feels more stable during sporting activities? ○ repaired knee ○ reconstructed knee ○ no difference
9. Which knee are you currently more confident about during sporting activities? ○ repaired knee ○ reconstructed knee ○ no difference
10. Which knee is your better knee overall? ○ repaired knee ○ reconstructed knee ○ no difference.
